# Climate analogues suggest limited potential for intensification of production on current croplands under climate change

**DOI:** 10.1038/ncomms12608

**Published:** 2016-09-20

**Authors:** T.A.M. Pugh, C. Müller, J. Elliott, D. Deryng, C. Folberth, S. Olin, E. Schmid, A. Arneth

**Affiliations:** 1 Institute of Meteorology and Climate Research, Atmospheric Environmental Research, Karlsruhe Institute of Technology, Kreuzeckbahnstrasse 19, 82467 Garmisch-Partenkirchen, Germany; 2 School of Geography, Earth & Environmental Science and Birmingham Institute of Forest Research, University of Birmingham, Edgbaston, Birmingham B15 2TT, UK; 3 Potsdam Institute for Climate Impact Research, PO Box 60 12 03, 14412 Potsdam, Germany; 4 University of Chicago and Argonne National Laboratory Computation Institute, Chicago, Illinois 60637, USA; 5 Columbia University Center for Climate Systems Research and NASA Goddard Institute for Space Studies, New York, New York 10025, USA; 6 Ecosystem Services and Management Program, International Institute for Applied Systems Analysis, A-2361 Laxenburg, Austria; 7 Department of Geography, Ludwig Maximilian University, 80333 Munich, Germany; 8 Department of Physical Geography and Ecosystem Science, Lund University, Sölvegatan 12, S-223 62 Lund, Sweden; 9 Department of Economics and Social Sciences, University of Natural Resources and Life Sciences, Vienna, Feistmantelstrasse 4, 1180 Vienna, Austria

## Abstract

Climate change could pose a major challenge to efforts towards strongly increase food production over the coming decades. However, model simulations of future climate-impacts on crop yields differ substantially in the magnitude and even direction of the projected change. Combining observations of current maximum-attainable yield with climate analogues, we provide a complementary method of assessing the effect of climate change on crop yields. Strong reductions in attainable yields of major cereal crops are found across a large fraction of current cropland by 2050. These areas are vulnerable to climate change and have greatly reduced opportunity for agricultural intensification. However, the total land area, including regions not currently used for crops, climatically suitable for high attainable yields of maize, wheat and rice is similar by 2050 to the present-day. Large shifts in land-use patterns and crop choice will likely be necessary to sustain production growth rates and keep pace with demand.

## Abstract (web summary)

Simulations of the impact of future climate change on crop yield vary considerably. Here, the authors use a climate analogue approach to estimate the response of maximum attainable yield to climate change and predict that large shifts in land use and crop choice would be required to meet demand.

## Graphical abstract

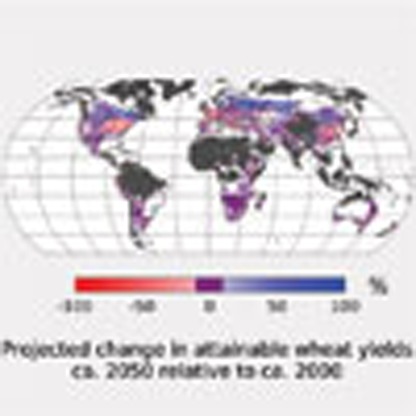

Between 1960 and 2000, global cereal production doubled, primarily through intensification of agriculture on current croplands and by the breeding of more productive crop varieties^1^. It is projected that global food production needs to increase by another 60–110% by 2050, to keep up with anticipated increases in human population and changes in diet^2^,^3^. Increases of this magnitude are possible through aggressive intensification in areas that are currently far below their potential^4^. However, several challenges exist relating to our ability to overcome economic, societal and environmental impediments to intensification^3^,^4^,^5^. To address these challenges effectively, and with a long-term outlook, it is necessary to understand how climate change is likely to impact the agricultural sector. Current expectations are that changes in climate over the next century will tend to decrease yields at lower latitudes and increase yields at northern latitudes^6^,^7^, but no consensus exists on the magnitude, timing and exact location of these changes.

Assessments of future food production under climate change have to-date relied on synthesis of crop-climate assessments at field and/or regional scales using models which vary from highly process-based to relatively empirical in formulation^6^,^8^. Both approaches can give important insights, but suffer also from significant limitations. Empirical models implicitly capture the effects of all relevant processes over the period and location for which they were parameterized, but they are often poorly tested for the conditions likely to become the future norm under climate change^8^,^9^, and are typically limited in their spatial coverage. Process-based models account explicitly for physiological mechanisms, and can thus capture emergent behaviour under novel conditions, but generally suffer from missing processes, such as heat stress and the coupling of transpiration to leaf temperature^8^,^10^. Many research groups now develop and operate global-scale crop-climate models, but the necessary data for model development, parameterization and evaluation is often not available at this scale^11^, and in most cases, global-scale assessments fail to account for farmer adaptation to climate change^7^,^9^. As a result, these models vary strongly in both the magnitude and overall direction of their yield projections, an effect which is especially pronounced across the tropics and arid regions^7^.

This uncertainty impedes understanding the scope of the societal challenge of climate change impacts on agriculture, particularly in developing countries where agriculture has larger shares in gross domestic product. Furthermore, large uncertainties in yield projections hinder our ability to assess the potential of various climate-change adaptation versus mitigation options; future changes in the productivity of different cropland areas will affect future land use, including societal decision-making on whether to, for example, reforest or intensify an area of cropland, or to extend cropland coverage^12^,^13^,^14^.

We use here a data-driven approach, utilizing observation-based estimates of current maximum-attainable yield with existing technology (hereafter referred to as attainable yield, see Methods section for definition)^4^ and climate analogues^15^,^16^, to assess the vulnerability of yields of the three major global cereal crops, wheat, maize and rice to climate change. Using future climate projections from five General Circulation Models (GCMs) that contributed to the Coupled Model Intercomparison Project Phase 5 (CMIP5) as part of the Fifth Assessment Report of the Intergovernmental Panel on Climate Change (IPCC)^17^, we identify present-day analogues of future climatic conditions, across the current global cropland area, with medium- (time window of 2041–2059, henceforth mid-century) and long-term (2081–2099, henceforth end-of-century) perspectives. Analogue climates are defined based on the accumulated annual sums of growing-degree-days (GDD) and precipitation, although we also test other variable combinations and seasonal averaging periods (Methods section). We make the assumptions that, given no changes in the global cultivar pool, the attainable yields of crops are purely a function of climate, and that the most effective yield-enhancing managements available are either spatially and temporally invariant, or also a function of climate; for instance, irrigation is unlikely to be widely available in very dry climates (neglecting possible finite fossil groundwater supplies or large rivers). Based on these premises, a yield which can be attained in the present day under a given climate can also be attained in the future, under an analogous climate, but at a different location. Areas with fundamentally unsuitable soils are avoided either by restricting projection of our results to current cropland area, or by masking with soil suitability information (Methods section). Our approach is thus independent of crop modelling methodologies typically used to project future yields, although, as for crop models, the results remain subject to uncertainties in terrain and soil type, particularly at the sub-grid scale. Further, it has important added value as, in addition to considering climatic effects on crop yields, it implicitly considers adaptation of management and crop type in line with current technology available for a given climatic environment. The technique allows us to (i) assess the change in attainable crop yield across the current global cropland area for each of maize, wheat and rice, (ii) identify areas where current dominant crops are likely to no longer be suitable in future, and (iii) to explore potential new growth locations for these crops.

We find widespread reductions in attainable yields of major cereals across a large fraction of current cropland by ca. 2050, but find that the total global land area climatically suitable for these crops remains largely unchanged, suggesting that in the absence of substantial advances in technology, it will be necessary to rethink crop choice and land-use patterns to achieve substantial production growth rates in the future. Our results not only improve confidence in the projections of vulnerability of the yields of cereal crops to climate change, but also point towards needs for knowledge on new technologies, and changes in management and genotypes.

## Results

### Effects on current cropland

By mid-century, following a strong climate change scenario (Representative Concentration Pathway, RCP, 8.5; Methods section) we find that most current wheat, maize and rice areas are within climatic conditions under which these crops are also cultivated today (Supplementary Fig. 1), although the analogues may be drawn from very distant locations. Analogues in the tropics, however, deteriorate greatly by end-of-century, especially for rice, as climates begin to emerge which are inconsistent with present-day cultivation of these crops. The results are qualitatively similar to results for previous assessments in which climate analogues were applied to investigate changes in biodiversity and natural vegetation, and which also showed poor present-day analogues for end-of-century climates in the tropics with strong climate change^15^,^16^.

**Figure 1:**
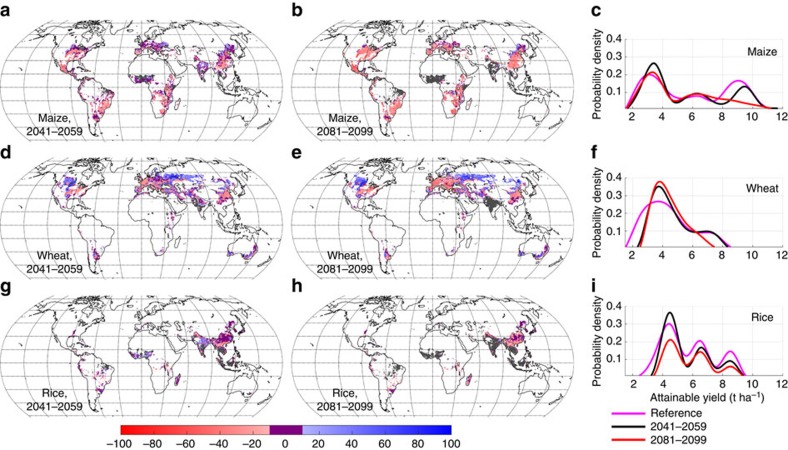
Change in attainable yield from the reference period to 2041–2059 and 2081–2099. Maps show percentage changes in attainable yield for for maize (**a**,**b**), wheat (**d**,**e**) and rice (**g**,**h**). Areas where yields change by 10% or less are marked in magenta. Areas with no present-day climate analogue are marked in grey (very few grid-cells). Yield changes are only shown for grid-cells which have a present-day climate analogue within the current harvested area of each crop, and where the current area devoted to that crop within the grid-cell exceeds 1,000 ha (ref. 35). Panels **c**,**f** and **i** show how the distribution of attainable yields at the grid-cell level is projected to evolve between the reference period (magenta line) and end-of-century (red line). Loss of area under the probability density curves is equivalent to the number of grid-cells for which no valid climate analogue can be found. Present-day attainable yields are obtained from ref. 4.

We find that large areas of current cropland are projected to experience strong reductions in attainable yields of major cereal crops over the next century (Fig. 1), indicating persistent vulnerability to climate change in the absence of significant advances in agricultural technology beyond that currently available. Our results are robust to the choice of GCM used to simulate climate, with agreement on the sign of yield change for at least four out of five GCM climates for the dominant spatial features in Fig. 1 (Supplementary Figs 2–7). Reductions are particularly seen in the number of grid-cells within the current harvested area that are projected to have a high attainable yield for maize and wheat (Fig. 1c,f). Vulnerable areas, which we define here as those showing a reduction in attainable yield >10% relative to the present-day reference data set^4^ and those which have no present-day analogue, vary by crop, but include some of the major regions of global food production (see below). Based only on areas where our results agree for at least four out of five GCM climates, our results indicate that already by mid-century 43, 28 and 40% of current global harvested area^18^ for wheat, maize and rice, respectively, is located in regions in which yields are vulnerable to climate change. In these regions, attainable yields are projected to either decline in the new climate, or no present-day analogue for the expected future climate can be found (Table 1). Vulnerable areas are not just located in low-producing regions; crops grown on areas designated as vulnerable by mid-century correspond to 43, 33 and 37% of the current global production of maize, wheat and rice respectively, increasing to ca. 74, 56 and 65% by end-of-century. If we consider all vulnerable areas indicated by at least one GCM climate, the vast majority of wheat, maize and rice production would be located in vulnerable areas by mid-century (Supplementary Table 1).

**Figure 2:**
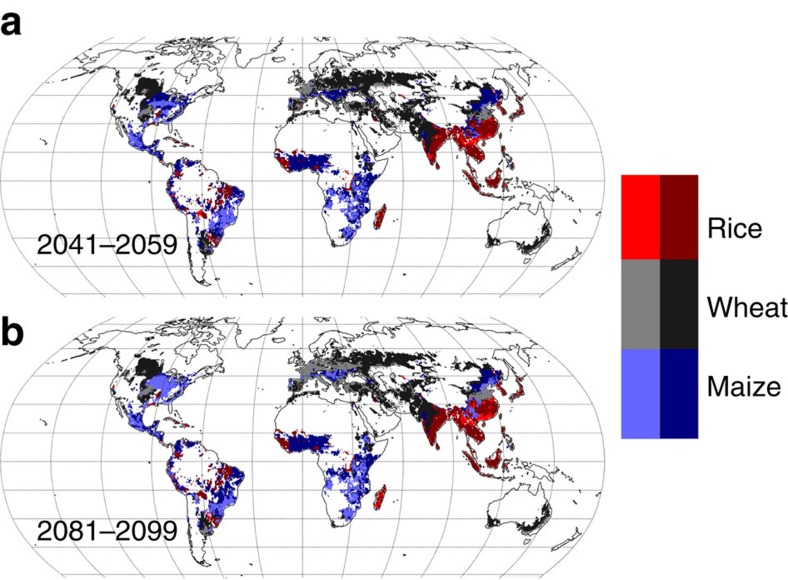
Yield vulnerability of dominant crop. Maps show grid-cells where the dominant crop by area is projected to undergo reductions in attainable yield of at least 10% by 2041–2059 (**a**) and 2081–2099 (**b**) relative to the reference period. The dominant crop is indicated by colour, with darker shades indicating that the attainable yield is maintained or increased, and lighter shades indicating areas in which it undergoes a decrease >10%. Only grid-cells where the sum of cropped area of wheat, maize and rice exceed 1,000 ha are shown^18^.

The spatial consequences of these changes in attainable yield for agricultural production can be appreciated by considering the effect on yields of the dominant crop at each location. Reductions in attainable yield of the dominant crop (by area) of >10% are projected across much of the eastern United States, Europe, Sub-Saharan Africa and temperate South America, and much of Eastern-Asia (Fig. 2). With the exception of regions with very strong management limitations, reductions in attainable yields are expected to generally imply reduction in actual yields unless measures can be taken to compensate by closing yield gaps (defined here as the difference between actual and maximum-attainable yields, given current technology). Highly developed countries, where yield gaps are already very small^4^, may thus face difficulties in sustaining current production without new technological interventions to increase attainable yield (for example, breeding novel crop cultivars). Such interventions could prove particularly challenging for maize and rice for which there has been little or no change in the genetic yield potential over recent decades^19^.

**Figure 3:**
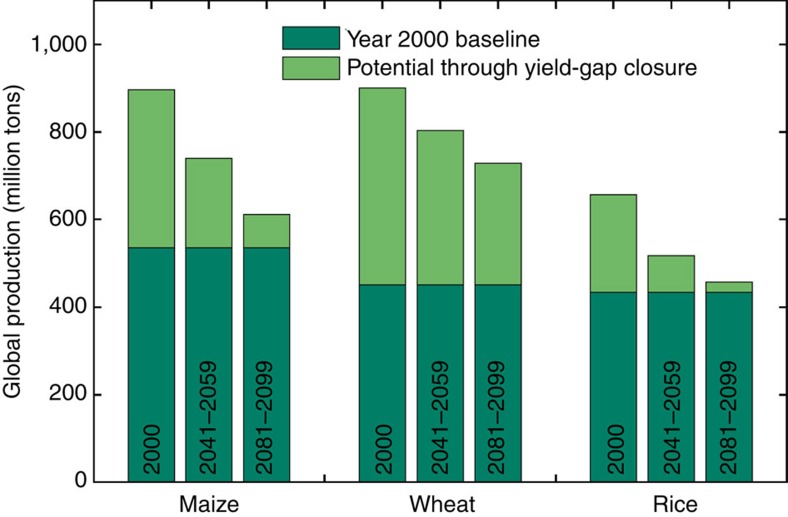
The effect of climate change on production increases through agricultural intensification. The potential increases in global production of maize, wheat and rice that could be achieved by closing the yield gap (difference between actual and attainable yields) on current cropland are plotted for baseline, mid-century, and end-of-century climates (light green bars). For reference, these bars are imposed on top of actual production for ca. year 2000 (dark green bars), as calculated based on actual yields from Mueller *et al*.^4^ and cropland area from Monfreda *et al*.^18^.

The effect of these changes in attainable yield on efforts to intensify cereal production will be particularly marked; there will be a greatly reduced capacity to intensify crop production beyond current levels through efforts to close the yield gap on existing croplands. It has been estimated that closing yield gaps in current climates could increase global production of maize, wheat and rice by 67, 100 and 52%, respectively, on their current harvested areas^4^. Based on end-of-century climate analogues, we estimate these potential increases over year 2000 yields to be only 14, 62 and 6% (Fig. 3), an average 57% reduction in global attainable yield growth potential. Intensification of production on current croplands therefore appears highly unlikely to be able to meet growing global demand over the next decades.

Although climate change stands to reduce production potential in many parts of the world, it nevertheless brings new opportunities in some regions. We identify large areas of current croplands where attainable yields increase substantially under climate change, with enhancements often exceeding 50% (Fig. 1). These areas are most prominent for wheat in the northern mid-latitudes, and include the large concentration of cropland in the central Canadian wheat belt, as well as western and central Russia. Assuming yield depressions from mismanagement, pests, diseases and other stresses are no higher in the target region than in the analogue region, this implies substantial increases in actual yields across these regions as a result of climate change.

### Changes in climatically suitable areas for cropping

**Figure 4:**
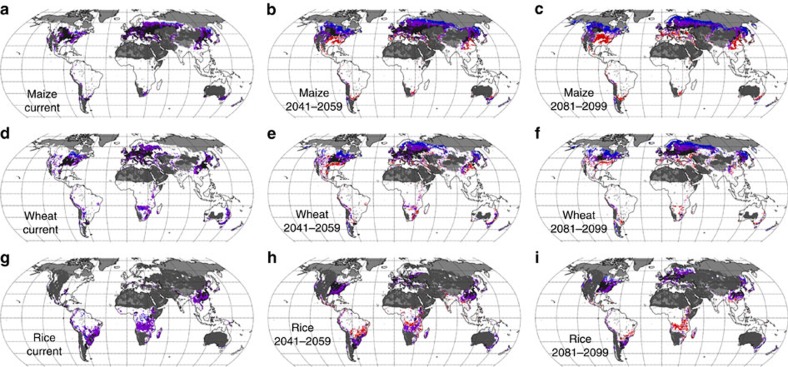
Areas with climate suitable to provide attainable yields greater than present-day median attainable yields. Present day median attainable yields are 8.1 t ha^−1^ for maize, 4.6 ton ha^−1^ for wheat and 5.2 ton ha^−1^ for rice. Note that climatically suitable does not necessarily mean economically or socially viable. Grid-cells are coloured black, purple, or blue when analogues produce attainable yields over the crop-specific thresholds for four out of five GCMs. Black shows grid-cells (0.5° × 0.5°) meeting the yield criteria which are currently heavily cropped (>50,000 ha harvested area over all crop types). Purple colours show grid-cells which are currently lightly cropped (1,000–50,000 ha harvested area). Grid-cells which have a current harvested area <1,000 ha are coloured blue. Red grid-cells show cropped areas which had an attainable yield over the threshold in the present day, but fell below this threshold in the future. White areas do not obtain the yield threshold. Panels **a**,**d** and **g** show the current situation^4^, **b**,**e** and **h** the situation in 2041–2059 based on climate analogues from the ensemble of climate models, and **c**,**f** and **i** the same for 2081–2099. Current cropland areas are taken from Monfreda *et al*.^18^. Areas with soils classed as fundamentally unsuitable for cropping over at least 90% of the 0.5° grid-cell are masked out in light grey, whilst areas with very low precipitation relative to the typical growing conditions of that crop are masked out in dark grey (Methods section).

Climate change can also increase the production potential of crops to levels of economic significance in regions which have hitherto been essentially uncropped. By expanding our analysis beyond the current harvested area of crops, we show that a large belt of land at high northern latitudes develops climates which are suitable for the production of wheat or maize with attainable yields by mid-century that are at least equivalent to the median attainable yield for that crop across current harvested area (Fig. 4); the area of land poleward of the 30th parallels climatically suitable for attainable yields above this threshold increases from 1.8 × 10^9^ to 2.1 × 10^9^ ha for maize and from 1.6 × 10^9^ to 1.7 × 10^9^ ha for wheat (Supplementary Table 2). Notably, the area of land climatically suitable for rice expands strongly to mid-century in the extratropics, but decreases in the tropics. Under RCP 8.5, the size of climatically high-yielding areas in the tropics collapses by end-of-century for all three crops, highlighting the importance of avoiding such a strong climate-change scenario. The nature of these results is qualitatively consistent with simple suitability indices based upon GDD alone, and with results from global crop models (Supplementary Note 1, Supplementary Figs 8-10). Nonetheless, whilst this global-scale analysis is indicative of increased climatic suitability for cropping in many areas that are not principally unsuitable from large-scale soil constraints^20^ (Methods section), we caution that because of its global scale it does not take account of local variations in soils or terrain properties which might adversely affect yields (although it is worth noting that poor soils can be remediated^21^). It also says nothing of whether it is economically viable to put in place the measures to achieve such yields in these locations. Ultimately, climatic suitability must be qualified by considering local-scale drivers.

### Influence of climate change scenarios

Although we base our results on a strong climate-warming scenario (RCP 8.5), large changes in attainable yield are apparent already by mid-century, when warming projections between RCPs have not yet strongly diverged^22^. Consequently, our results for mid-century are qualitatively similar, although of slightly lower magnitude, when calculated for the low climate change RCP 2.6 scenario (Supplementary Fig. 11). In fact, estimated mid-century impacts on attainable yield, both positive and negative, are much stronger than most of the projections of actual yield synthesized in the IPCC Fifth Assessment Report^6^. This reflects the fact that when climate is the main limiting factor for yields, then changes in climate will have a greater effect, and implies that greater intensification likely brings greater vulnerability of yields to climate change. This is in line with previous findings that weather variability often is the main driver of yield variability in highly productive areas, but not necessarily in areas of low productivity^23^.

### Comparison against crop models

The pattern of attainable yield changes in Fig. 1 are very similar to those reported for actual yields in previous assessments^24^, with the exception of the positive changes in West Africa and India at mid-century, which we discuss further below. We further compare the changes in attainable yield produced by our data-driven method, with those of an ensemble of gridded global crop models recently used to assess the impact of climate change on crop yields as part of the ISI-MIP project^7^ (Supplementary Figs 12 and 13). The simulations we compare against here are conceptually similar to the climate analogue approach in that they use fixed atmospheric CO_2_ mixing ratios [CO_2_] from 2000 onwards, however they differ in the fact that most models use fixed present-day fertiliser levels rather than unlimited nutrients, and in that the simulations allow unlimited irrigation, which may not be possible in some low-rainfall areas. Although the model-based climate-impact ensemble projections have a broad spread, the qualitative pattern of yield changes is very similar to the analogue method, with yield losses generally in the tropics and arid areas and increases in high latitudes. Notable differences between the model ensemble and climate analogue method arise for maize and rice in India and tropical West Africa. Here the models tend to show yield decreases, whilst the analogue approach suggests increases, however, we have lower confidence in yield projections in these locations as they show sensitivity to the analogue variable choice (Supplementary Note 2). The models are also much more pessimistic regarding changes in wheat yields in the mid-latitudes, and especially across central-Asia. These more pessimistic projections from the models may result from limited or absent consideration of climate adaptation^7^. In reality, farmers will change their preferred cultivars, growing seasons and other management actions, as climate evolves. The analogue method implicitly accounts for cultivar adaptation within the bounds of the pool of globally-used cultivars, and for changes in growing seasons and other managements, possibly leading to positive changes in attainable yield in some regions where strongly negative effects have previously been expected. As there are many differences in methodology between the crop models and our analogue method, it is not possible to definitively attribute these different regional responses to adaptation. However, the comparison nonetheless demonstrates that inclusion of adaptation within the bounds of current technology does not fundamentally alter the global picture of yield change over the 21st century. Further comparison with models is provided in Supplementary Note 3.

### Source of analogues

A feature of the analogue method is that it identifies present day regions whose agricultural practices and crop varieties may hold lessons for other regions in the future, and thus where efforts should be made to maximize technology and knowledge transfer, that is, transferring existing crop varieties from the analogue to the target location, although we do not imply that the analogue approach can be used to derive explicit technology transfer pathways. It is not feasible to catalogue all potentially important analogue locations in a global analysis. However, we show some examples in Supplementary Fig. 14, and summarize the general situation using the mean latitudinal distance from which analogues are drawn (Supplementary Table 4). Mid-century analogues for cropland poleward of the 30th parallels are drawn, in the mean, from regions 18°, 13° and 22° latitude closer to the equator for maize, wheat and rice, respectively, and change relatively little between mid- and end-of-century, as analogues have already largely reached the extent of current harvested area of the crop in question by mid-century.

## Discussion

Our analogue-based method only considers climate-induced changes in yield, but increased [CO_2_] has been found to directly enhance photosynthesis in C3 crops, whilst also promoting greater water use efficiency across all higher plants^25^. These [CO_2_] effects may offset some of the strong negative effects of climate change on attainable yields. A meta-analysis of field experiments suggests a yield enhancement of ca. 8% for non-water-stressed C3 crops for a doubling of [CO_2_] from pre-industrial levels, although this number disguises huge variability in responses^26^. For an RCP 8.5 scenario this would imply a yield increment due to [CO_2_] of +6% by 2050, and +18% by 2100, relative to 2000, although the response may be larger in some water-limited regions. Changes of this magnitude only marginally reduce the large vulnerable areas of wheat and rice by mid-century, although by end-of-century they would reduce substantially vulnerable areas of wheat and rice in East Asia (Supplementary Fig. 15). Attainable yields of maize, a C4 crop, are not expected to be affected by increased [CO_2_], although actual yields of all crops may be substantially enhanced in areas where water stress is limiting growth^25^,^27^. Simulations from the ISI-MIP ensemble show enhancements of wheat and rice yield due to CO_2_ fertilization ranging from 4 to 80% (Supplementary Table 3), illustrating the huge uncertainty in projections of CO_2_ fertilization, however, direct comparison is complicated by the differing assumptions on nutrient availability and climate adaptation between the models (see Supplementary Note 3 and Supplementary Figs 16 and 17).

The analogue method employed here has a number of limitations and uncertainties. The approach assumes that management practices for maximizing yield can be directly transferred between grid-cells. In most cases we expect this to be true, as socio-economic limitations do not apply in the context of attainable yields. However, there may be some circumstances where this concept fails due to specificity of managements to terrain or soil types. In areas with low precipitation, the method will tend towards more positive projections if analogues are drawn from grid cells in which sources of irrigation water are disconnected from local precipitation, that is, regions with large fossil groundwater abstractions or supplied by large rivers. These projected yields remain attainable, but water resources will not allow them to be reached on a large scale unless the grid cell for which yields are being projected also has access to such a water resource. We find no clear evidence of our projections for current cropland area being notably influenced by this effect, but if they were the effect would be to make our conclusions of vulnerability more conservative. It is very relevant, however, for projections of suitable future cropland area. As a result, we have marked such areas in Fig. 4, and excluded them from the other calculations in this analysis (Methods section). Importantly, our projected changes in attainable yields must not be taken as being indicative of changes in rainfed yields in regions where irrigation water is potentially available, but is not utilized on socio-economic grounds; the relation between attainable and rainfed yields is non-linear. Although we mask fundamentally unsuitable soils (Methods section), we do not include variations in soil quality across current cropland area in our analogues. Soil is an important determinant of crop productivity, and thus some of the increases in yields suggested by our method may be limited by poor soil quality. There is a particularly high uncertainty in assessing the importance of soil quality in high northern latitudes, where soil core data are very scarce^28^. Here there is a risk that the analogue method tends to overestimate attainable yields on unimproved soils. We suggest, however, that because properties such as soil nutrient content, organic carbon and pH can be remediated through amendments and management, soil quality is generally a limitation on actual, rather than on attainable, yields. Our conclusions are robust to the choice of analogue variables, with simulations including killing degree days (degree-days above a crop-specific threshold for heat damage) and radiation, excluding precipitation, or limiting to current growing seasons, giving extraordinarily consistent results for yields, despite some variation in the location of the analogue climates (Supplementary Note 2).

We contend that early investment in regions that are likely to be climate analogues in the future could serve a dual benefit, both helping to alleviate any deficiencies in food production in these regions today, whilst also serving to inform, and thereby speed-up, climate adaptation in other locations in the future. This is particularly important when complications to the simple ‘transfer' perspective arise where for instance tropical analogues are found for temperate grid-cells, and differences in, for example, day-length requirements may require identification and transferal of adapted germplasm, a process with potentially long lead-times^19^,^26^. However, while the climate analogue approach could be a (biophysically based) tool to assess technology transfer, the specific boundaries of such an analogue framework would need to be evaluated with observations of cross-location performance of crop and soil management technologies before it could be operationally applied in this context.

Even with the assumption of adaptation within current technology inherent in our approach, climate change is projected to decrease production potential, and therefore likely actual production, from major cereal crops across the tropics and much mid-latitude cropland already by the middle of the 21st century. Our results thus provide an independent line of evidence qualitatively supporting, and thereby increasing confidence in, projections from crop models. In the absence of successfully breeding new crop cultivars to suit warmer climates, attempts to intensify crop production across much of existing cropland are likely to be frustrated by climate change, requiring compensation by other means. We conclude that much more efficient and sustainable production increases are likely to be achieved by altering crop choice and the location of cropland itself, to best take advantage of changes in maximum-attainable yields resulting from climate change. However, we caution that socio-economic constraints, along with any unmanageable aspects of local terrain and soil, will influence whether the climatic potential calculated here can be realised; detailed local-scale assessments will thus be necessary to assess this. Such land-use alterations do not necessarily imply extensification (that is, increasing the area under cultivation). However, changing the distribution of agricultural area might in some regions lead to dramatic changes in land use that will face political, social and cultural impediments if, for instance, some regions need to become net food importers or to change traditional diets. A drastic shift in distribution of cropland would likely also have very serious consequences for other ecosystem services such as carbon storage provided by non-agricultural lands where such a reconfiguration may take place^29^, although these consequences might be mitigated by the regeneration of areas of abandoned cropland in the long-term. Rather than being able to rely principally on continued intensification on existing croplands, as is often a major assumption of economic models^13^,^14^, the need to increase crop production is therefore likely to present a major land-use, trade and economic dilemma over the coming decades. Furthermore, for achieving attainable yields projected by the analogue approach, our results imply the need to transfer technologies and knowledge over large distances, demonstrating that there is yet another important dimension in the agricultural challenge that requires a global perspective; not only trade and markets, but also production knowledge needs to be globalized.

## Methods

### Attainable yields data set

Present-day maximum-attainable yields used in this analysis are obtained from the data set created by Mueller *et al*.^4^. Mueller *et al*. divided harvested area of each crop into 100 equal-area bins based on GDD and annual precipitation. They then calculated the attainable yield as the 95th percentile of yields within each climate bin, after exclusion of outliers. As such, attainable yields represent a maximum level consistently achievable in a climatic zone, given current technology, and not physiological potential yields.

### Identification of climatic analogues

We define climate analogues using an approach previously used to identify novel and disappearing climates and changes in vegetation carbon under climate change^15^,^30^. The method is modified here for use with crops. Difference between climates is calculated using Standardised Euclidean Distance (*SED*),


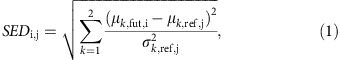


where *μ*_k,ref_ is the mean of climate variable *k* over the reference period (1981–1999), and *μ*_k,fut_ the mean over the future period (2041–2059 or 2081–2099), *σ*_k,ref_ is the s.d. of the interannual variability over the reference period. The letter *i* refers to the primary grid-cell, and *j* to any other grid-cell which *i* is being compared against.

Temperature and water availability are fundamental constraints on crop growth. Since growing seasons can be adapted to changing climate, we consider annual (rather than seasonal) sum of GDD (base temperatures, *T*_b_, of 0, 8 and 5 °C for wheat, maize and rice, respectively^31^) and mean precipitation as climatic variables *k*(1) and *k*(2). This maintains consistency with the methods used to derive the attainable yield data set^4^. Precipitation is included because areas with very low rainfall may not have the option of irrigation (that is, the attainable yield is a rainfed yield). Seasonal timing of precipitation is not considered critical, as water may be stored for irrigation later in the year. More discussion of the effect of analogue variable choice is provided in Supplementary Note 2 and Supplementary Fig. 18. *SED* is calculated individually for every crop type. For every 0.5° × 0.5° grid-cell, *i*, *SED* is calculated between the future climate in that grid-cell, and reference climates in all grid-cells, *j*, including at least 1,000 ha of that crop^18^. We note that harvested area has expanded substantially for some crops since Monfreda *et al*.^18^ was published^32^, but this data set remains the *de facto* standard and maintains consistency with Mueller *et al*.^4^, from whom we have taken the present-day attainable yield data. As changes in cropped area are much smaller than for harvested area, this should have minimal influence on our results.

For any given grid-cell *i*, the grid-cell *j* under reference period climate which returned the minimum value of *SED* was chosen as the best present-day climate analogue for the future climate at location *i*. The attainable yield for the crop in question, as calculated by Mueller *et al*.^4^ for ca. year 2000, from the chosen reference-period grid-cell *j* was then taken as the attainable yield for that crop in grid-cell *i*. In the results presented herein, we used the mean of the projected yield across the five best analogues to each grid-cell *i*. Note that when we test the production potential of grid-cells outside the current harvested area of a crop (Fig. 4), we only allow analogues to be drawn from within the current harvested area. Values of *SED* and changes in attainable yield were calculated individually for bias-corrected daily climate data from each of 5 Global Climate Models (GCMs): IPSL-CM5A, HadGEM2-ES, GFDL-ESM2M, MIROC-ESM-CHEM, NorESM2 (ref. 33). Presented values represent the mean of results from those 5 GCMs, unless stated otherwise. Calculations presented herein were made based on RCP 8.5 (ref. 34), but were also performed for RCP 2.6 to test sensitivity to moderate climate change.

### Calculation of threshold *SED*

To capture values of *SED* which are so high as to indicate that there is no climate in the reference period (within the current harvested area of the crop in question) from which to draw an analogue to the future climate, we calculate a threshold value of *SED*, *SED*_t_, above which the analogue is considered too weak for conclusions regarding attainable yield to be drawn. To calculate *SED*_t_, we randomly draw two sets of 19 individual years from the period 1981 to 2010 and assign one as climate ‘ref' and the other as climate ‘fut', and then calculate *SED* as per Equation E1. We thereby calculate for each grid-cell a value of *SED* for the case when climates in each location are drawn from the same distribution, which we term *SED*_D_. We then define *SED*_t_ as the characteristic maximum value of *SED*_D_ (excluding outliers) by calculating its 95th percentile across the current harvested area of each crop. We thus follow the premise that for an analogue to be valid for projecting crop yield, the value of *SED* for that analogue must be similar to the *SED* calculated between two climates drawn from the same distribution. Taking the mean value of *SED*_t_ per crop across the 5 GCM climates generates values of 0.7, 0.7 and 1.0 for maize wheat and rice, respectively. For simplicity, we take the most conservative of these, *SED*_t_=0.7, as our baseline, however the projections of attainable yield are extremely robust to this choice (Supplementary Fig. 19). This definition of *SED*_t_ closely reflects the calculated gradients in this variable between typically cropped and non-cropped areas (Supplementary Fig. 1). We also use these two sets of 19 random years from the same climate distribution to show that the climate analogue method calculates only minimal changes in yield when the climate is unchanged (Supplementary Note 4, Supplementary Fig. 20).

### Analysis of climatic potential for cropland expansion

In considering areas of potential cropland expansion (Fig. 4 and Supplementary Table 2), areas with soil fundamentally unsuitable for cropping were masked out based on the GAEZ v3.0 database^20^: we combined soil suitability indices at a resolution of 10 arc minutes for excess salts, oxygen availability, rooting conditions, toxicities and workability, and excluded any areas that were not marked as minimal or moderate constraints in any of the five indices. Conditions relating to nutrients were neglected, as it is considered that fertilization would be required to achieve attainable yields in any case. To mask out areas with very low rainfall drawing analogues with regions with substantial fossil groundwater or non-local irrigation water sources (that is, water sources independent of grid-cell annual mean precipitation), we calculated the 5th percentile of the distribution of reference period annual mean precipitation across the current rainfed area of each crop^35^. We then excluded from the cropland expansion analysis all grid-cells with annual mean precipitation below this level.

### Crop modelling

Comparisons against Global Gridded Crop Models (GGCMs) used data from 6 GGCMs (LPJ-GUESS, LPJmL, pDSSAT, PEGASUS, EPIC, GEPIC)^7^. These GGCMs were run with the same climate data used for the *SED* calculations herein for the period 1971–2099, and simulated maize, wheat and rice at 0.5° × 0.5° resolution for all land grid-cells (no rice simulations from PEGASUS). GGCM set-ups were not harmonized except for climate and [CO_2_]. Simulations were made both with transient [CO_2_], and with [CO_2_] fixed to 369 ppmv from year 2000 onwards. Here we compare our climate-analogue-derived attainable yields with modelled yields under full irrigation.

### Data availability

The authors declare that all data supporting the findings of this study are available within the article and its Supplementary information files or are available from the corresponding author upon request.

## Additional information

**How to cite this article:** Pugh, T. A. M. *et al*. Climate analogues suggest limited potential for intensification of production on current croplands under climate change. *Nat. Commun.* 7:12608 doi: 10.1038/ncomms12608 (2016).

## Supplementary Material

Supplementary Information Supplementary Figures 1 - 20, Supplementary Tables 1 - 4, Supplementary Notes 1 - 4 and Supplementary References

## Figures and Tables

**Table 1:** Portion of current global production and harvested area in vulnerable or no-analogue zones for at least four out of five GCM climates.

	**2041–2059**		**2081–2099**	
	Vulnerable*	No analogue	Vulnerable*	No analogue
*Current production % ( × 10^6^ t)*				
Maize	39.0 (209)	3.5 (18)	67.7 (363)	6.3 (34)
Wheat	29.8 (134)	3.3 (15)	38.5 (174)	17.1 (77)
Rice	13.4 (58)	23.7 (103)	17.8 (77)	47.4 (206)
				
*Current harvested area % ( × 10^6^ ha)*				
Maize	35.2 (44)	8.0 (10)	54.1 (67)	17.2 (21)
Wheat	22.6 (44)	5.4 (10)	27.7 (54)	17.6 (34)
Rice	11.4 (13)	28.9 (34)	11.7 (14)	60.5 (71)

^*^Reduction in attainable yield >10%.
