# Supplementary material for: Climate analogues suggest limited potential for intensification of production on current croplands under climate change
Source: Nat Commun. 2016 Sep 20;7:12608. doi: 10.1038/ncomms12608 (PMC5136618; doi:10.1038/ncomms12608)
Supplement: Supplementary Information — Supplementary Figures 1 - 20, Supplementary Tables 1 - 4, Supplementary Notes 1 - 4 and Supplementary References [file ncomms12608-s1.pdf]

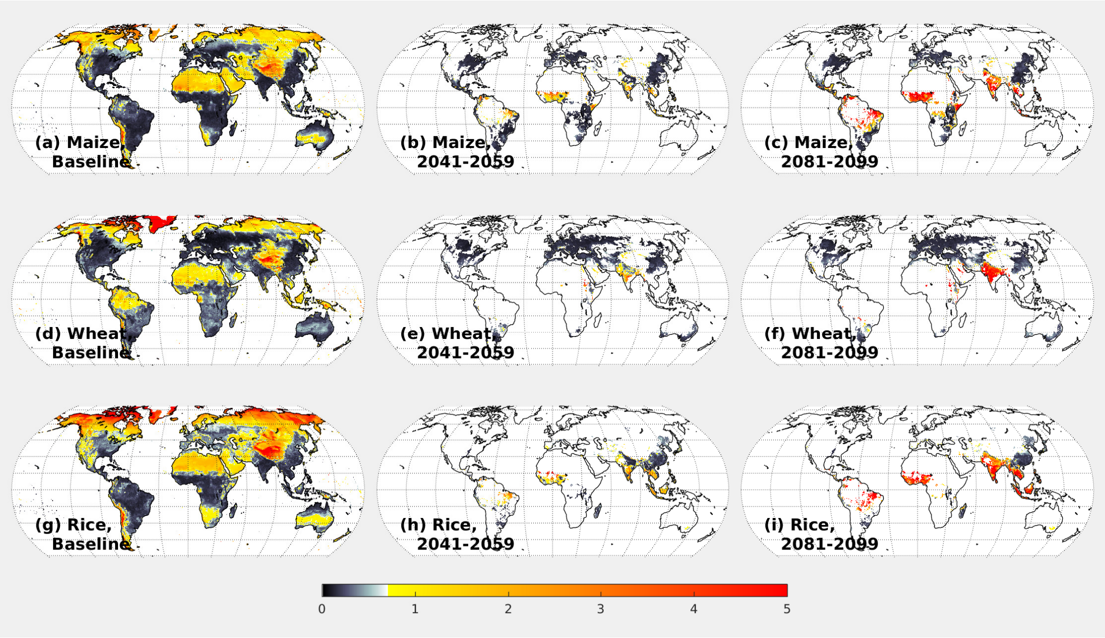

Supplementary Figure 1. **Ensemble mean *SED*.** Maps displayed for maize (a, b, c), wheat (d, e, f) and rice (g, h, i), for baseline climate, 2041-2059 and 2081-2099. The calculations for the crops differ in the base temperature used for the growing degree days calculation and in the crop areas used to draw the present-day analogues from (see Methods). Values are only shown for grid-cells which contain more than 1000 ha harvested area of the respective crop, except for the baseline climate, which is shown unmasked to illustrate the generally strong spatial gradients in *SED* around SED_t. Areas with *SED* greater than 0.7 are considered to have only poor climate analogues in the reference period (1981-1999) (see Methods), and are marked in yellow-red shades.

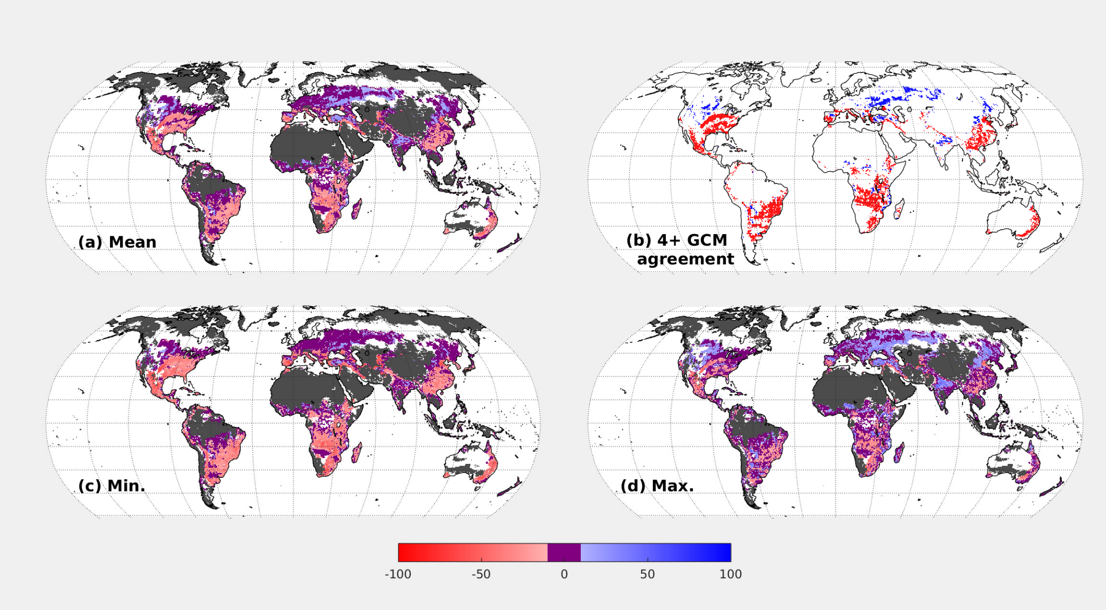

Supplementary Figure 2. **Change in attainable yield of maize for 2041-2059 relative to the reference period.** Panel a shows the ensemble mean, panel c the ensemble minimum, and panel d the ensemble maximum. The grid-cells for which climate analogues from at least 4 GCMs show agreement in the sign of attainable yield change are marked in panel b; red indicates agreement in a reduction of attainable yield, blue an increase in attainable yield. Areas where yields change by 10% or less are marked in magenta. Areas with no present-day climate analogue are marked in grey. Results are only shown for grid-cells which have a present-day climate analogue within the current harvested area of maize. Attainable yields are obtained from Mueller et al.[1].

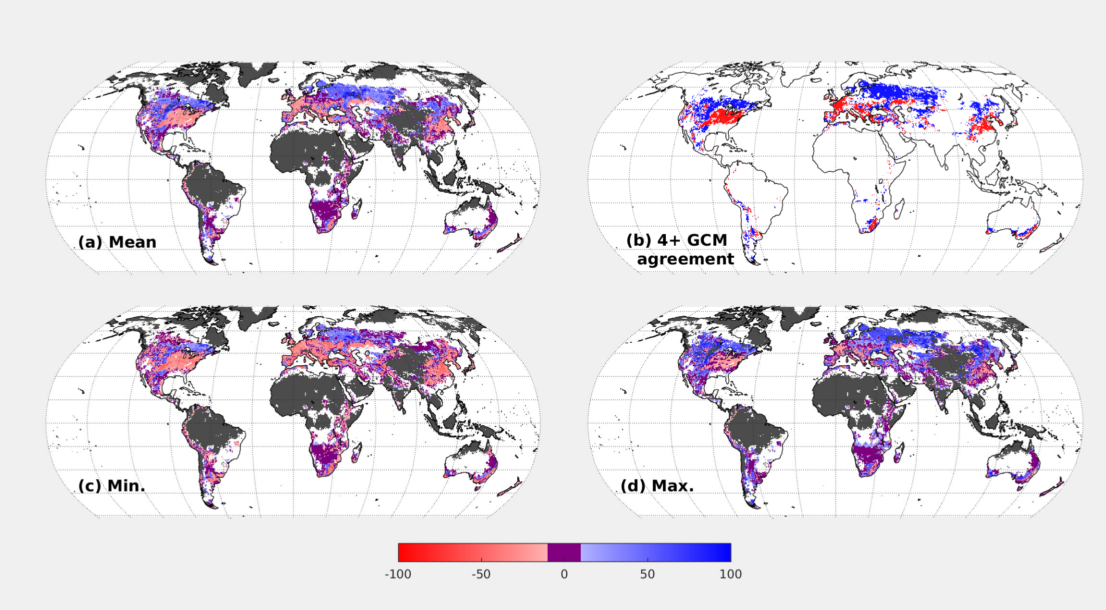

Supplementary Figure 3. **Change in attainable yield of wheat for 2041-2059 relative to the reference period.** Description as for Supplementary Figure 2.

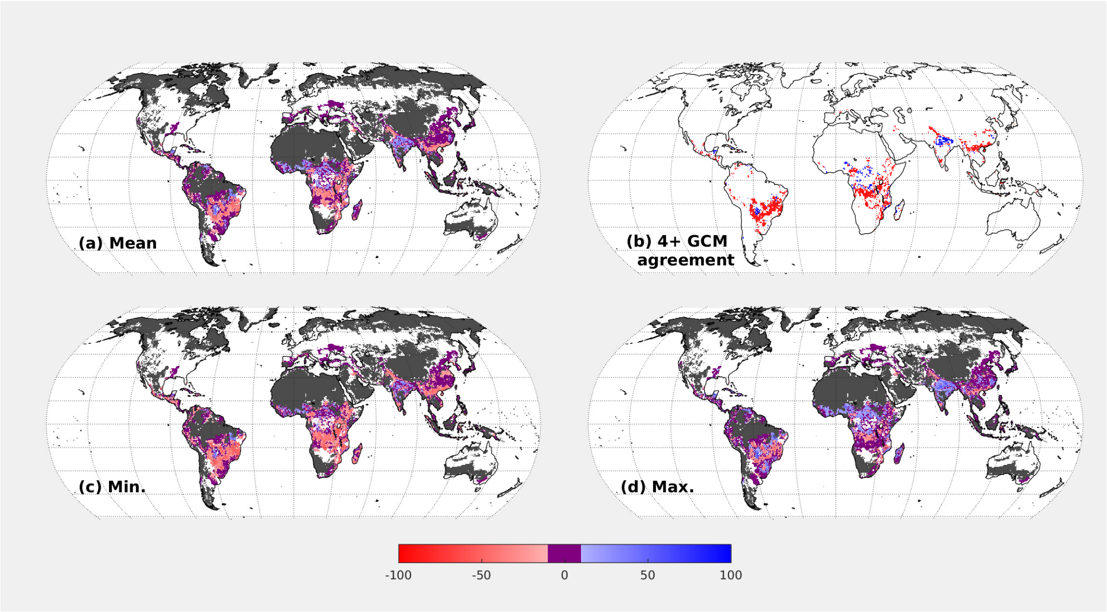

Supplementary Figure 4. **Change in attainable yield of rice for 2041-2059 relative to the reference period.** Description as for Supplementary Figure 2.

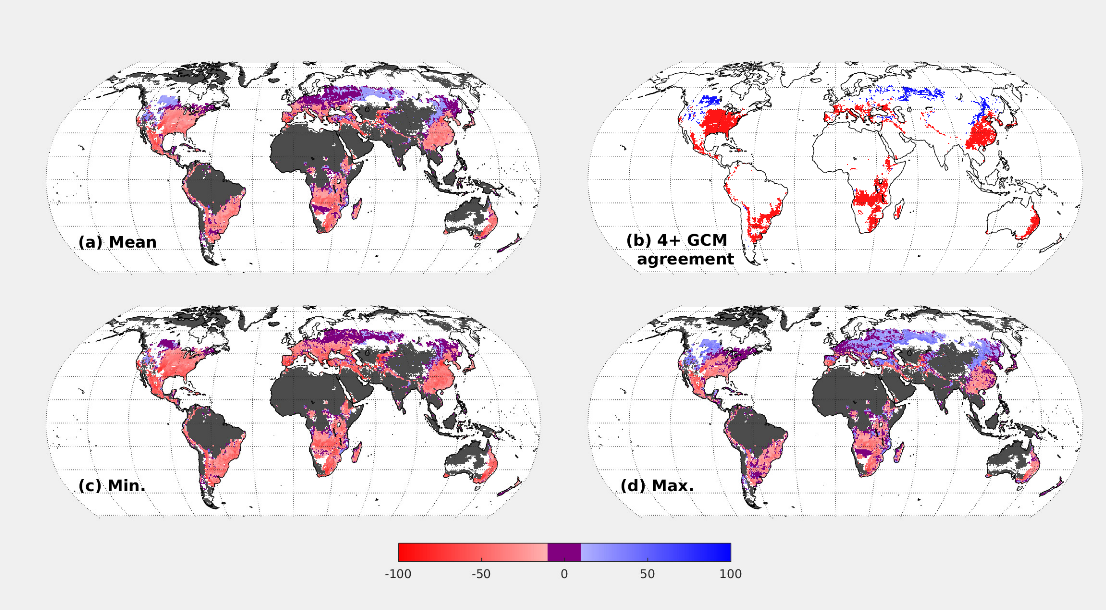

Supplementary Figure 5. **Change in attainable yield of maize for 2081-2099 relative to the reference period.** Description as for Supplementary Figure 2.

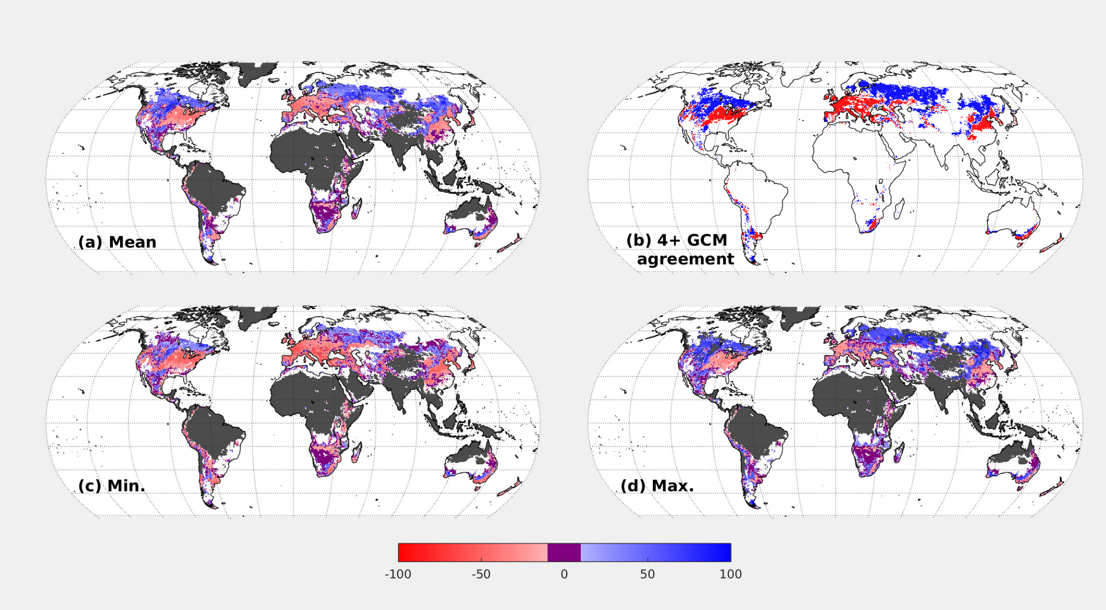

Supplementary Figure 6. **Change in attainable yield of wheat for 2081-2099 relative to the reference period.** Description as for Supplementary Figure 2.

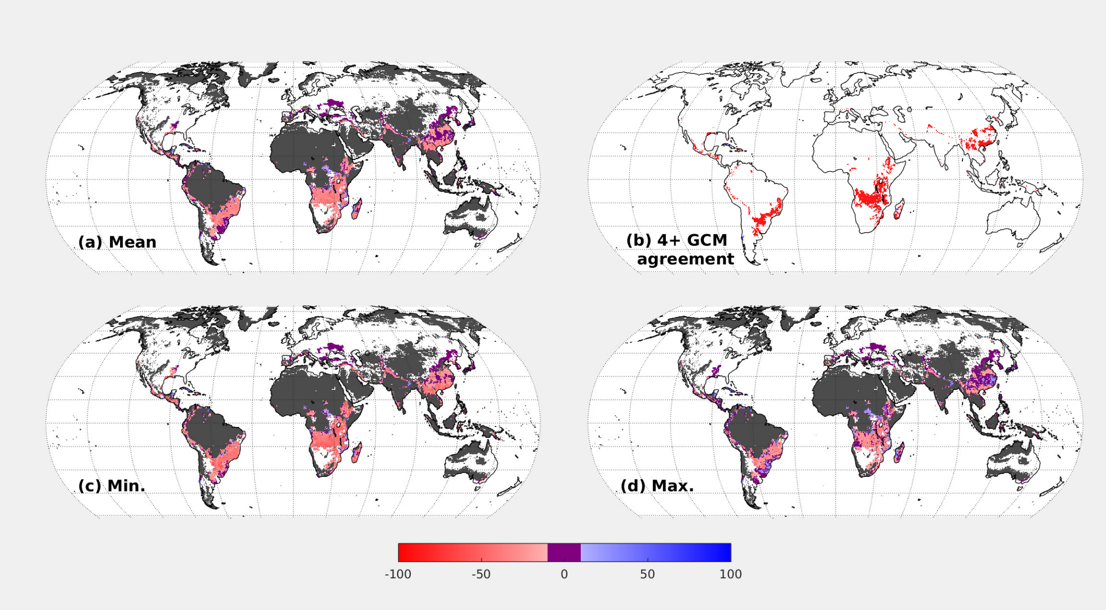

Supplementary Figure 7. **Change in attainable yield of rice for 2081-2099 relative to the reference period.** Description as for Supplementary Figure 2.

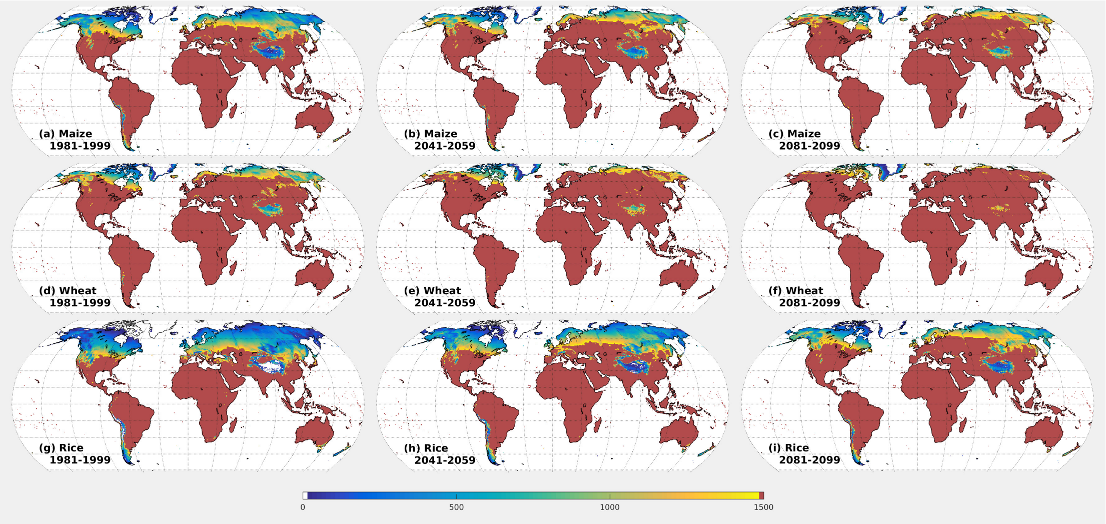

Supplementary Figure 8. **Calculation of location of climatic suitability threshold based on GDD alone.** GDD is calculated using base temperatures from Bondeau et al.[2], and all areas with an annual GDD sum of at least 1500 degree-days, a globally-applicable threshold for maturity corresponding to the chosen base temperatures[2], are marked in red. Comparison can be drawn with Fig. 4 for assessing climatic suitability in high latitudes.

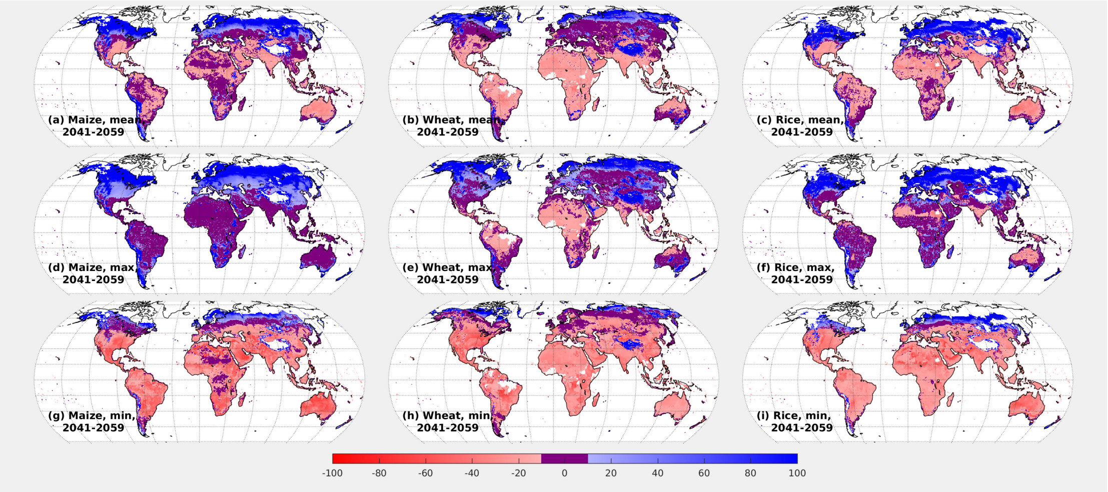

Supplementary Figure 9. **Change in simulated irrigated yield for an ensemble of six Global Gridded Crop Models for the period 2041-2059 relative to 1981-1999.** The models are EPIC, GEPIC, LPJmL, PEGASUS, pDSSAT, LPJ-GUESS. The results shown are for simulations described in ref.[3] in which atmospheric CO_2 mixing ratio was fixed at 368.9 ppmv from the year 2000 onwards, and for which the crops were fully irrigated (i.e. closest analogy to the analogue method available from the simulations), and represent averages over five GCMs (same set and bias-correction as used for the analogue calculations herein). The top row shows the mean results from the six GGCMs, the middle row show the most positive changes in yield across the GGCM ensemble, and the lower row the most negative changes. Colouring is as for Fig. 1. Climate changes follow the RCP 8.5 scenario. Results displayed for all ice-free land area with simulated irrigated yields over 1 ton ha^{-1}.

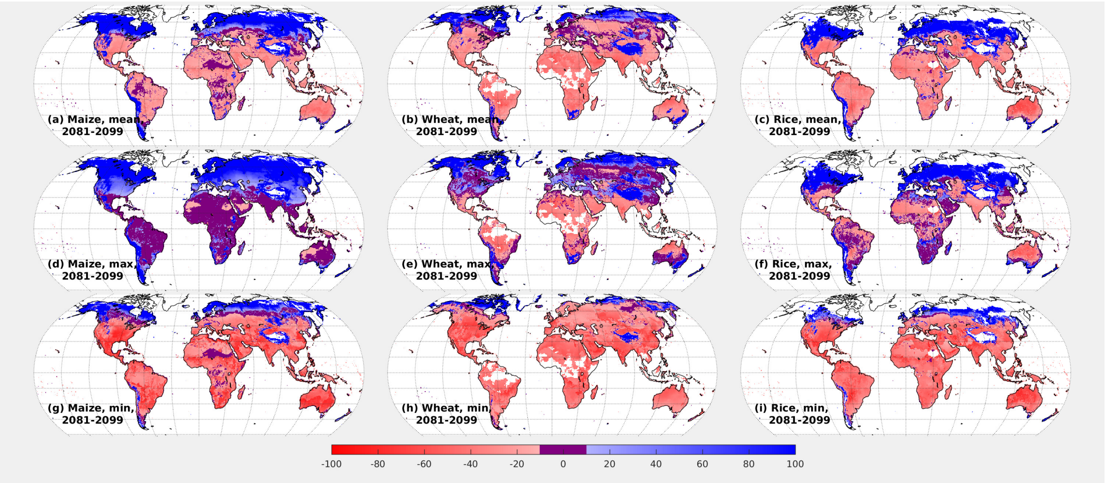

Supplementary Figure 10. **Change in simulated irrigated yield for an ensemble of six Global Gridded Crop Models for the period 2081-2099 relative to 1981-1999.** Description as for Supplementary Figure 9.

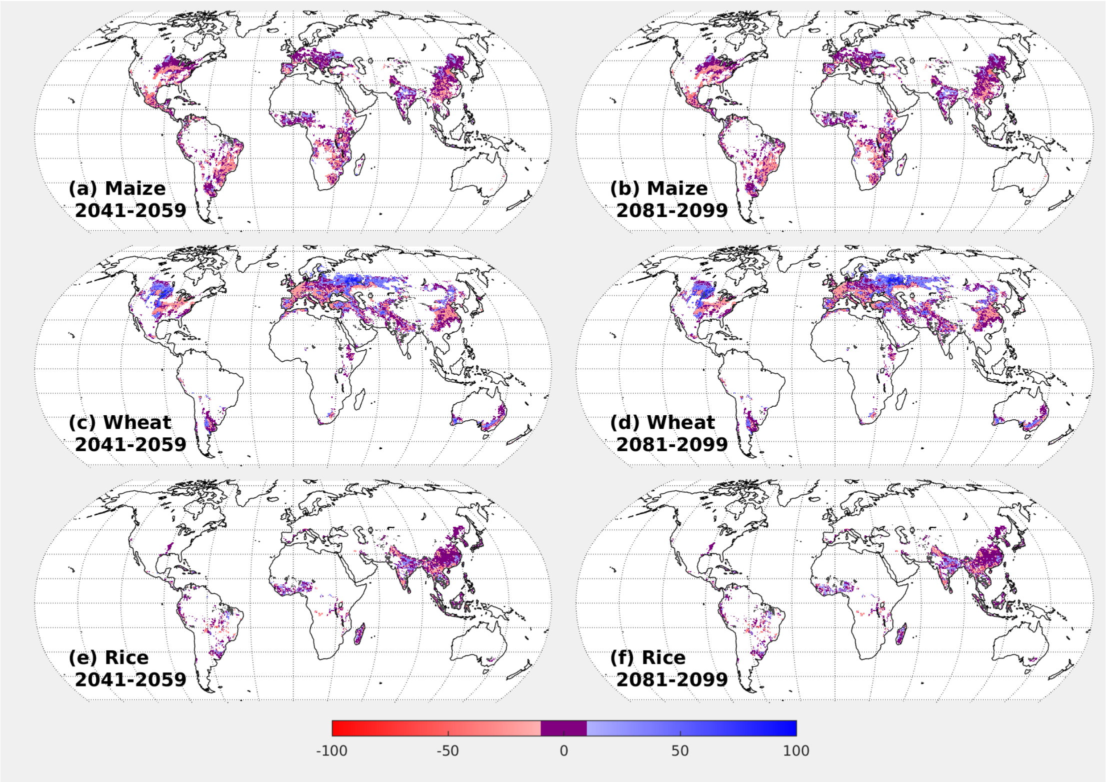

Supplementary Figure 11. **Change in attainable yield from the reference period to 2041-2059 and 2081-2099 for RCP 2.6.** Description as for Fig. 1

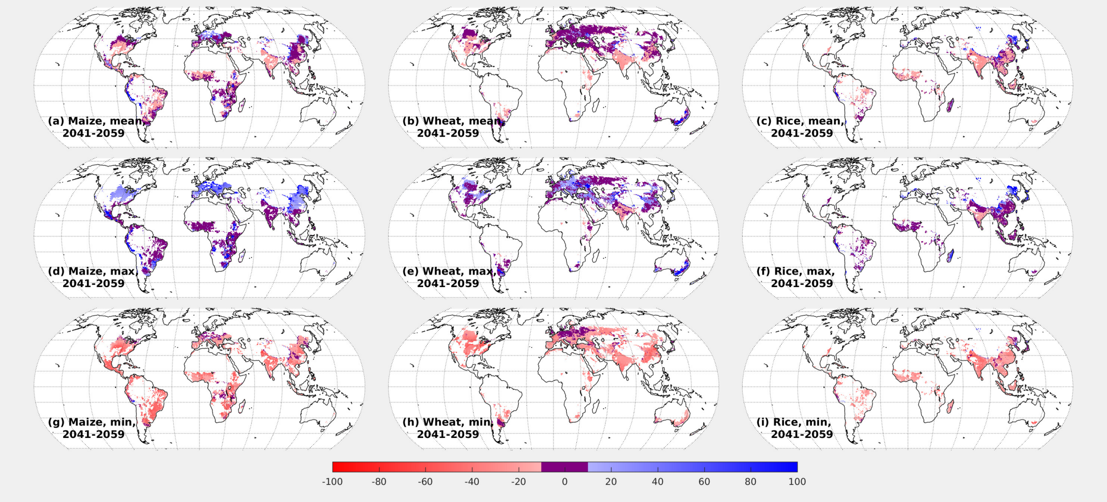

Supplementary Figure 12. **Change in simulated irrigated yield for an ensemble of six Global Gridded Crop Models for the period 2041-2059 relative to 1981-1999.** Data is identical to in Supplementary Figure 9, but results are only shown for the current harvested area.

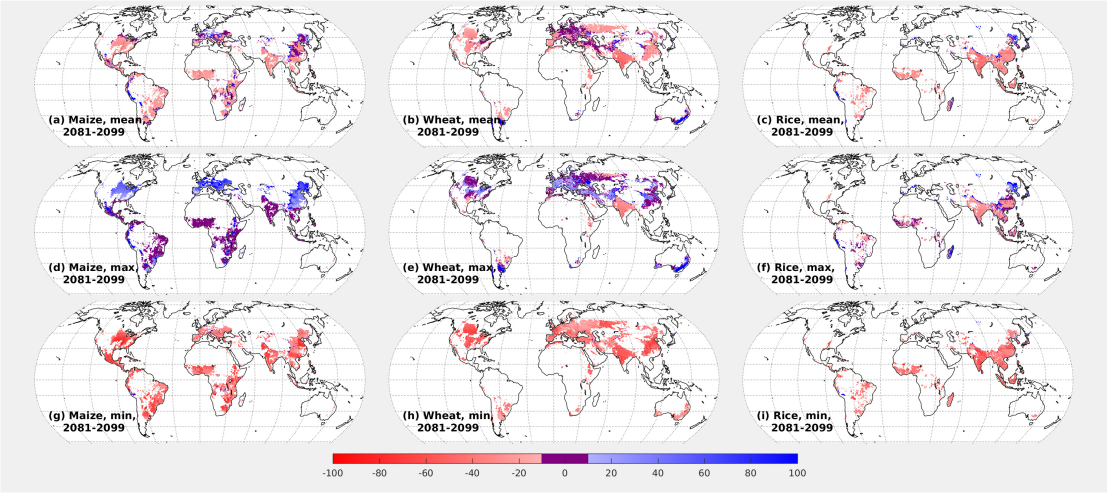

Supplementary Figure 13. **Change in simulated irrigated yield for an ensemble of six Global Gridded Crop Models for the period 2081-2099 relative to 1981-1999.** Data is identical to in Supplementary Figure 10, but results are only shown for the current harvested area.

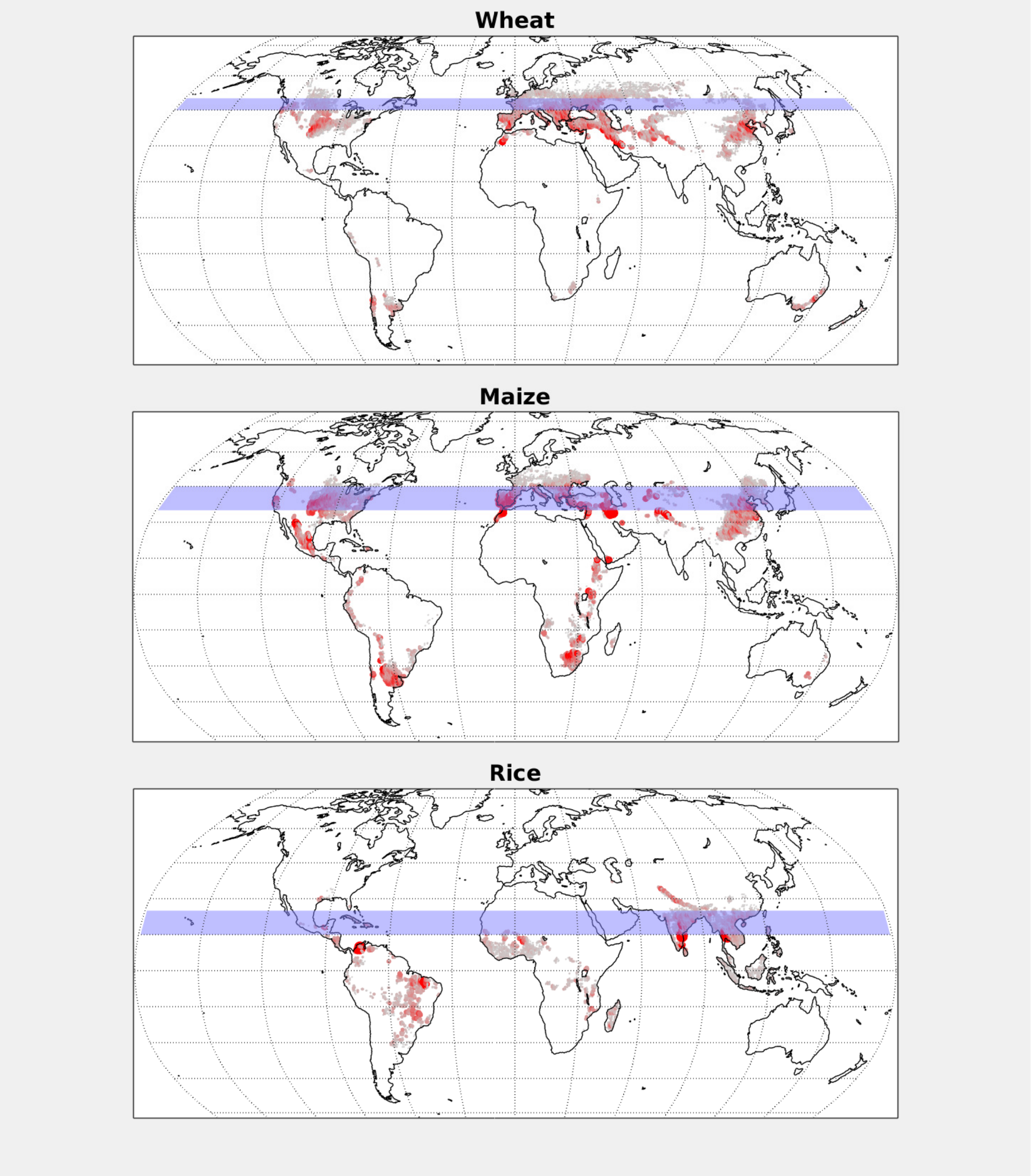

Supplementary Figure 14. **Example of source of climate analogues.** Coloured dots show the sources of climate analogues for selected regions and crops (shaded blue areas) for the period 2041-2059. Only analogues corresponding to grid-cells including harvested area of the relevant crop are shown. Dots are coloured (grey to red) and sized, according to the number of times that grid-cell is used as an analogue. Analogues for all 5 GCM climates are plotted, although the spread of results is similar for each GCM.

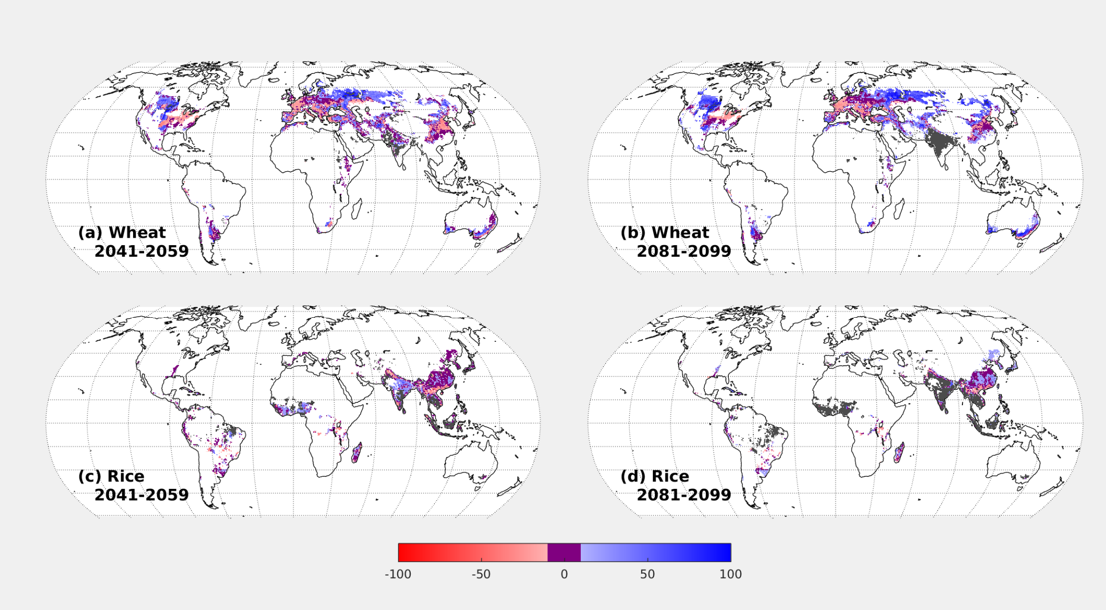

Supplementary Figure 15. **Change in attainable yield from the reference period to 2041-2059 and 2081-2099 adjusted for CO_2 fertilisation.** Maps for wheat and rice as for Fig. 1 except that changes in attainable yield for wheat and rice have an additional +6% for 2041-2059, and +18% for 2081-2099 included in the displayed values, to give an indication of the effect of CO_2 fertilisation.

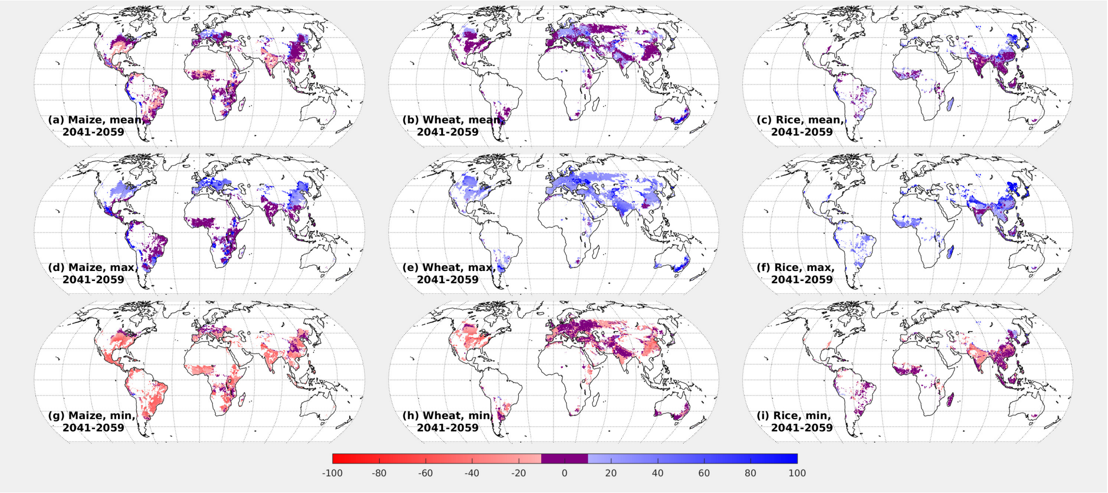

Supplementary Figure 16. **Change in simulated irrigated yield for an ensemble of six Global Gridded Crop Models for the period 2041-2059 relative to 1981-1999 under evolving [CO_2].** Description as for Supplementary Figure 12, but with atmospheric CO_2 mixing ratio allowed to follow the RCP 8.5 scenario from 2000-2059.

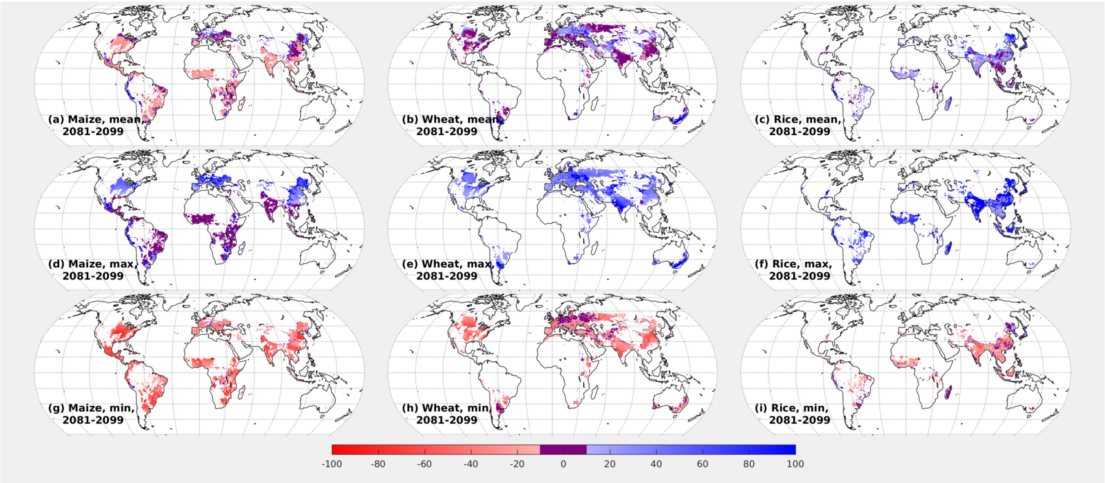

Supplementary Figure 17. **Change in simulated irrigated yield for an ensemble of six Global Gridded Crop Models for the period 2081-2099 relative to 1981-1999 under evolving [CO_2].** Description as for Supplementary Figure 13, but with atmospheric CO_2 mixing ratio allowed to follow the RCP 8.5 scenario from 2000-2099.

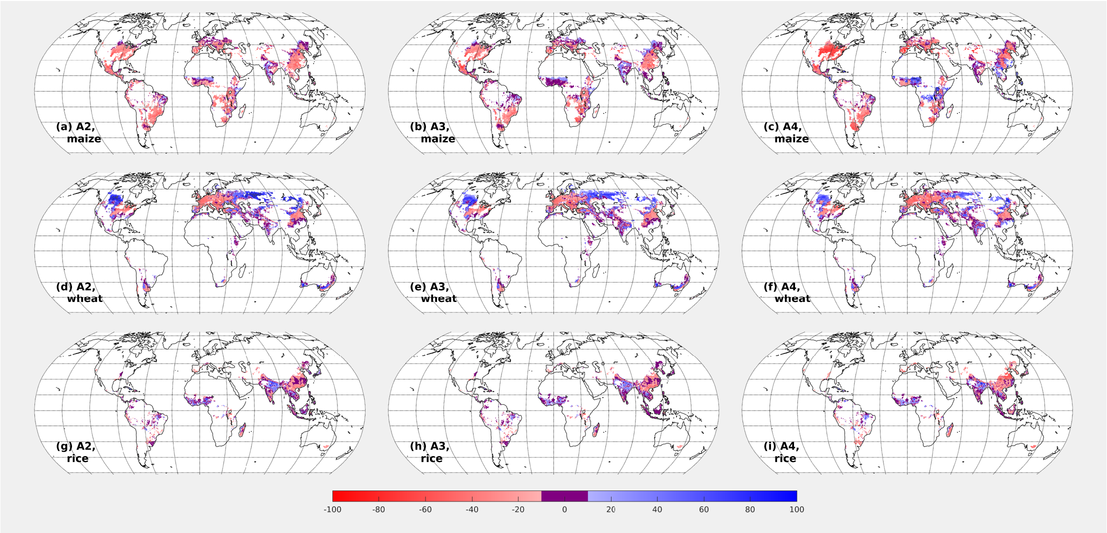

Supplementary Figure 18. **Change in attainable yield as calculated using three alternative sets of analogue variables.** Values shown are percentages for 2081-2099, relative to present day. Variable combination A2 is shown in panels a, d and g; A3 in panels b, e and h, and A4 in panels c, f and i. Poor analogues are not masked. See Supplementary Table 4 for explanation of the variable sets.

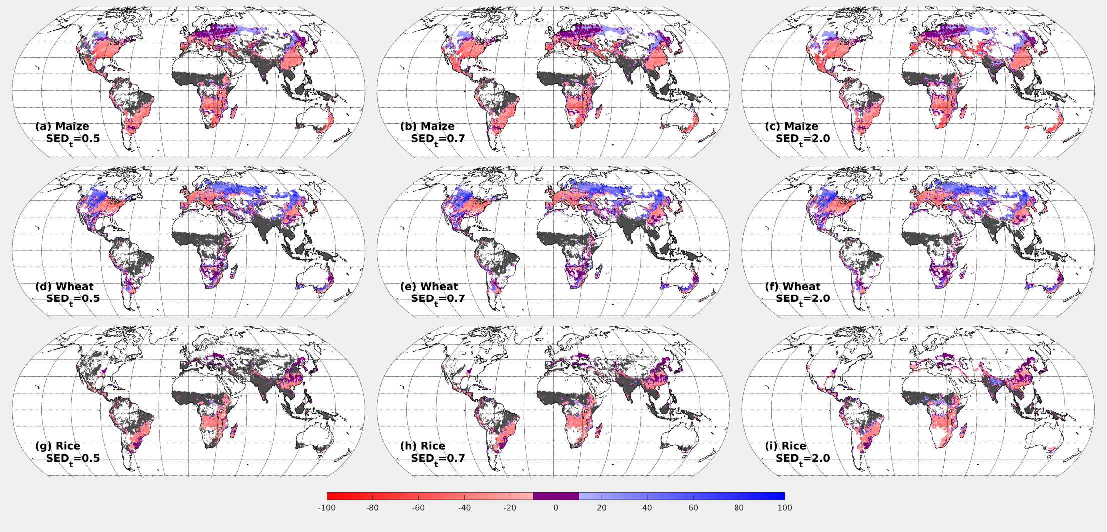

Supplementary Figure 19. **Effect of *SED* threshold on projections of future attainable crop yield.** Maps are created as for Fig. 1 for the change from the reference period to 2081-2099 for maize (a, b, c), wheat (d, e, f) and rice (g, h, i). Results for SED_t=0.5 are presented in the left column, and for SED_t=2.0 in the right column. Results for SED_t=0.7 (standard) are provided in the centre column for ease of comparison.

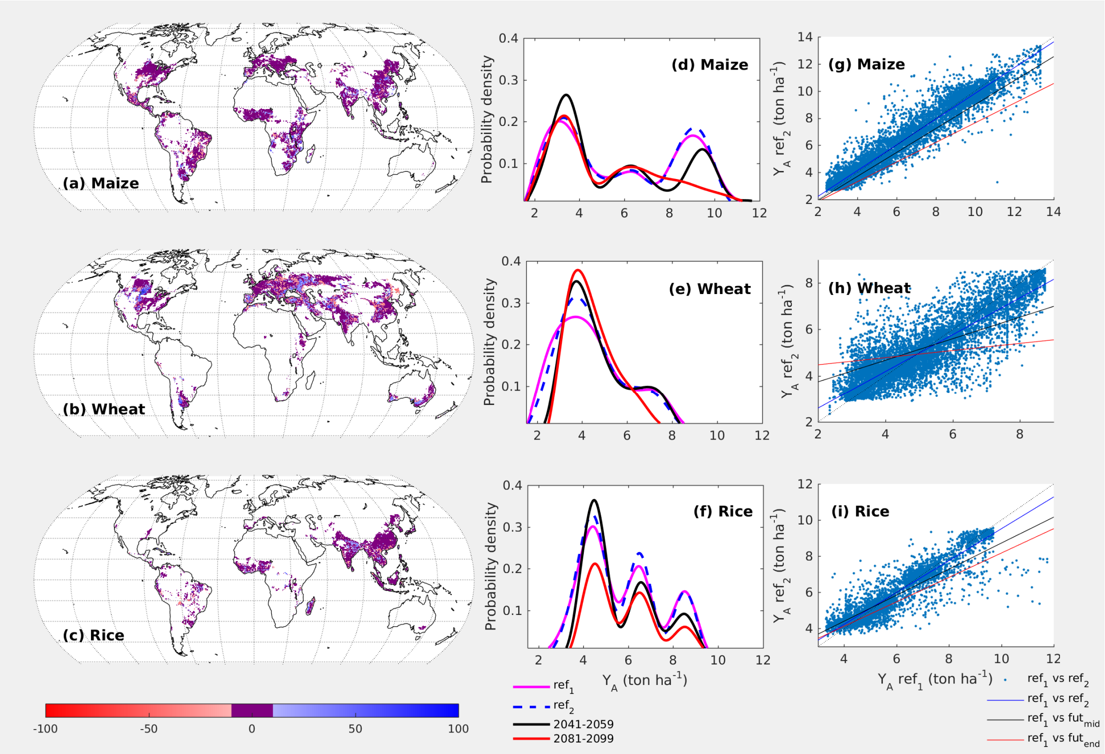

Supplementary Figure 20. **Test of the climate analogue method for a baseline climate.** The analogue method was applied to two random non-continuous samples of 19 years (ref_1 and ref_2) extracted from the period 1981-2010. Panels a-c show the projected change in attainable yields. Panels d-f show the distribution of attainable yields for the ref_1 (magenta line) and ref_2 (blue dashed line) climates, as well as the projections for mid- and end-of-century (from Fig. 1). Panels g-i show the correlation of attainable yields between ref_1 and ref_2 climates. Linear correlations are plotted as solid lines for ref_1 vs ref_2 (blue), ref_1 vs mid-century (black), ref_1 vs end-of-century (red). The 1:1 line is plotted as black dots.

Supplementary Table 1. Portion of current global production and harvested area in vulnerable or no-analogue zones for at least 1 GCM climate.

	Current yield % (×10[6] tons)			
	2041-2059		2081-2099	
	Vulnerable[1]	No analogue	Vulnerable[1]	No analogue
Maize	**65.5** (351)	**6.0** (32)	**88.4** (474)	**7.8** (42)
Wheat	**53.6** (242)	**14.4** (65)	**52.9** (238)	**23.5** (106)
Rice	**31.5** (137)	**38.1** (165)	**41.7** (181)	**50.1** (217)
	Current harvested area % (×10[6] ha)			
Maize	**59.6** (74)	**14.4** (18)	**75.8** (94)	**20.5** (25)
Wheat	**42.9** (83)	**17.8** (35)	**42.8** (83)	**26.7** (52)
Rice	**26.1** (30)	**46.2** (54)	**29.8** (35)	**64.3** (75)

[1] Reduction in attainable yield greater than 10%.

Supplementary Table 2. Total land surface area where the climate is suitable to provide attainable yields above thresholds of 8.1, 4.6 and 5.2 ton ha^{-1} for maize, wheat and rice respectively.

	Current	2041-2059	2081-2099
Global			
Maize	1.92	2.10	1.76
Wheat	2.12	1.95	1.76
Rice	1.69	1.81	1.51
Extratropics (>30° lat.)			
Maize	1.80	2.06	1.73
Wheat	1.61	1.74	1.67
Rice	0.33	1.00	1.16
Tropics (<30° lat.)			
Maize	0.13	0.04	0.03
Wheat	0.51	0.21	0.09
Rice	1.36	0.81	0.35

Areas with unsuitable soil or low precipitation are excluded (see Methods). Units are $\times 10^9$ ha.

Supplementary Table 3. Enhancements of non-water limited yields in the global gridded crop model ensemble relative to the baseline period due to the effect of CO_2 fertilisation.

	Change in yield relative due to CO_2 (%)					
	EPIC	GEPIC	LPJ-GUESS	LPJmL	pDSSAT	PEGASUS
Wheat						
2041-2059	11.0	2.5	26.3	11.5	8.4	20.1
2081-2099	15.6	4.3	50.23	19.1	15.9	30.5
Rice						
2041-2059	17.2	6.9	48.5	21.1	16.0	n/a
2081-2099	22.9	13.9	80.4	39.6	28.4	n/a

Supplementary Table 4. Summary of analogue variable combinations tested and the latitudinal distance of analogues from the target grid-cell.

Code	Analogue variables	Annual or growing-season sums?	$D_{lat,end,30}$ (°)		
			Maize	Wheat	Rice
A1	GDD, precipitation	Annual	-19.3 (15.3)	-14.2 (11.8)	-21.7 (17.8)
A2	GDD	Annual	-11.7 (10.2)	-9.1 (9.2)	-15.7 (13.2)
A3	GDD, precipitation, KDD	Annual	-18.4 (16.0)	-12.3 (12.6)	-13.8 (11.6)
A4	GDD, precipitation, KDD	Growing season	-14.0 (11.5)	-13.1 (11.6)	-13.6 (10.7)
A5	GDD, precipitation, KDD, incoming shortwave radiation at land surface	Growing season	-18.4 (16.0)	-12.3 (12.6)	-13.8 (11.6)

Values in parentheses are standard deviations.

Supplementary Note 1

Cross-comparisons of potential high-yielding area change

We cross-compare our results with two alternative methods of projecting changes in high yielding area. We first compare with GDD sum thresholds, which have previously been used to estimate climatic suitability[6]. We take the GDD sum threshold of 1500 GDD for suitability along with the corresponding base temperatures of 0° for wheat, 5° for maize and 10° for rice, from Bondeau et al.[2], as these numbers are optimised for a global approach. The GDD sums, capped at 1500 degree-days and averaged over the five GCMs, are presented in Supplementary Figure 8. The patterns correspond closely to those presented in Fig. 4, giving confidence that the analogue method is not being overoptimistic in terms of potential areal expansion on a climate basis.

We also compare our results in Figure 4 with those from the global crop model ensemble (Supplementary Figures 9, 10). We cannot usefully recreate Figure 4 from the crop model results as the ISI-MIP simulations use widely differing management intensities (fertilisation, other aspects of agricultural intensity) for different regions of the world. For threshold-based comparisons of absolute yields these spatially-differing management intensities would lead to misleading maps. However, analysis of the relative actual yield changes shows that by the end of the 21st century under RCP 8.5 wheat yields are often only simulated to improve relative to the present day in very high latitude or altitude locations. I.e. climate change is so strong that climate-related gains are only found in climates which are currently very cold. Often these locations are ones masked out in our analysis of cropland expansion due to highly unsuitable soils. In contrast simulated maize yields typically show a strong positive change across relatively lower latitudes and altitudes than wheat. This behaviour is consistent with the results of the analogue method displayed in the threshold-based approach in Fig. 4.

Supplementary Note 2

Sensitivity to analogue variable

In order to establish the robustness of our results to the choice of analogue variable, we repeated the calculations with a variety of different combinations of climatic analogues, as summarised in Supplementary Table 4. The sum of Killing Degree Days (KDD) was defined as the accumulation of degree days above extreme temperatures (T_e) of 26°C, 29°C and 34°C for wheat, maize and rice respectively[4]. In calculations including KDD, the daily increment of GDD summations was capped at T_e - T_b. Crop-specific growing seasons were defined according to ref.[1]. SED_t was recalculated for each combination of variables.

The choice of analogue variables had often a substantial influence on the location from which analogues were drawn, in particular affecting the latitudinal distance from the target grid-cell from which analogues tended to be drawn. To summarise the changes in this latitudinal distance we calculated the mean absolute latitudinal distance (D_{lat}) of the analogue from the target grid-cell as,

$$D_{\text{lat}} = |L_{\text{anal}}| - |L_{\text{tar}}|\,,$$

where L_{tar} is the latitude of the target grid-cell, and L_{anal} the latitude of the grid-cell from which the analogue is draw. We use here values calculated for the end of the century

and taking the mean over target grid-cells poleward of 30° latitude to illustrate the differences in analogue location due to analogue variable choice ($D_{lat,end,30}$)

Only using annual GDD as an analogue variable (A2) substantially reduced the latitudinal distance from which the analogues were drawn, with $D_{lat,end,30}$ reducing from -14° - -22° to -9° - -16° (range over three crops), when compared to the standard analogue calculations (GDD and precipitation, A1). The inclusion of annual precipitation reduces the tendency of analogues to focus along latitudinal lines following equator-pole temperature gradients. Inclusion of annual KDD (A3) has a similar, although less marked, effect, as areas with high annual KDD tend to be at lower latitudes.

We chose to use annual sums for the analogue variables in order to not constrain crops to always be grown in the same season, i.e. to allow for adaptation to changes in climate patterns. However, in order to test whether conditions outside of the main growing season unduly influence the results of the analogues, we also carried out analogue calculations restricted to summing analogue variables over current main growing seasons (A4). We find broadly similar values for $D_{lat,end,30}$ under this method. Finally, we also tested the importance of changes in incoming shortwave radiation (A5), but once again found limited sensitivity of the analogue calculations to this constraint, probably because changes in radiation are related to changes in temperature and precipitation (through cloud cover).

Although the choice of analogue variables induced some variation in the sources of the analogues, the calculated projected changes in attainable yield proved extremely consistent, regardless of the method used (Supplementary Figure 18). Qualitatively, the results for yield change generated by methods A1-A3 are almost identical. When including the growing season constraint we found a tendency to slightly more extreme yield change projections, for instance for maize in North America and rice in eastern China, however notable changes in the direction of the result were only seen in parts of West Africa for maize, and southern India for rice. Tracking the source of the analogues for these regions reveals that including the growing season constraint shifts gives analogues from broadly the same regions, but also brings in a large group of analogues from north-east India for rice, and north-west India for maize, which bring down the yield projections. Yield projections for these parts of West Africa and India must therefore be considered to have a lower degree of confidence than for other regions.

Supplementary Note 3

Comparison of analogue crop projections and global crop models

To increase the robustness of our conclusions, we compare the changes in potential yield produced by our data-driven method, with those of the gridded global crop models (GGCMs) recently used to assess the impact of climate change on crop yields[3] (Supplementary Figures 12 and 13). These simulations used full irrigation of crops, and fixed [CO_2] from 2000 onwards, making them conceptually similar to the analogue calculations, although differences in treatment of fertilisation between the models mean that these results cannot be regarded as true potential yields. Although the models show a broad spread of quantitative changes in their projections, the qualitative pattern of yield changes is very similar to our climate analogue method, reinforcing the general pattern of yield losses in the tropics and arid areas, but increases in the high latitudes. Notable differences between the model ensemble and climate analogue method arise for maize and rice in India and tropical west Africa, where the models tend to suggest yield decreases; this pattern is reversed in the analogue projections, although these are also

the areas where the choice of analogue variable causes most uncertainty (see above), reducing confidence in projections in these regions. The models are also much more pessimistic regarding changes in wheat yields in the middle latitudes, and especially across Central Asia. These more pessimistic projections from the models may result from the lack of cultivar adaptation, which is common to all but two of these models (LPJ-GUESS and PEGASUS). Failure to adapt cultivars to climate means that growing season length will reduce, and consequently the projected yield will drop. In reality however, farmers will change their preferred cultivars as climate evolves. The analogue method implicitly accounts for cultivar adaptation within the bounds of the pool of globally-used cultivars, leading to more positive changes in attainable yield than if such adaptation was neglected.

The climate analogue approach may also produce different results to GGCMs because it does not identify individual cropping seasons, but rather characterises annual climate conditions. This annual climate characterisation is intentional, as it means that analogue choice is not limited to particular growing-season windows, which would effectively bar the selection of analogues from different hemispheres, or from regions where the wet season arrives at a different time of year. It also has the effect, however, of implicitly assuming adaptation of planting dates. This differs from the approach in most GGCMs (excluding GEPIC and PEGASUS) in which the timing of the main cropping season was assumed not to change. This level of implicit adaptation of planting dates may also contribute to more positive projections in the analogue method for some regions.

It is not possible to definitively allocate these differences in yield projections between models and the analogue method to cultivar or growing season adaptation, because of possible limitations in the attainable yield dataset in poorly-developed regions. The maximum-attainable yields from Mueller et al.[1] were calculated by dividing the global cropland into 100 bins with different characteristic combinations of precipitation and GDD. The 95th percentile of observed yields within a bin (after accounting for outliers) was then taken as the maximum-attainable yield. In most climate bins it is to be expected that there is sufficient well-managed cropland that this is robust. However, it is possible that in some tropical regions, climate bins may include very few intensively-managed croplands; parts of India and tropical West Africa may be candidates for such regions, leading the analogue approach to overestimate yield increases in these areas. However, given the wide variety of locations from which climate analogues are typically drawn, spanning multiple continents (Supplementary Figure 14), we expect that any such potential biases do not substantially influence our results.

GGCM simulations were also conducted using [CO_2] which evolved after 2005 following the RCP 8.5 scenario, thereby providing an alternative method with which to assess the potential importance of CO_2 fertilisation. There are however, substantial uncertainties in the modelling of the CO_2 fertilisation effect on crop yields, with inclusion of CO_2 fertilisation being found to greatly increase the uncertainty of model projections[3]. In these simulations, projections for non-water-limited maize were not modified by [CO_2], as maize is a C4 crop for which the sensitivity of photosynthesis to [CO_2] is saturated at levels far below those of the present atmosphere. It is noted, however, that for actual yields, CO_2 fertilisation is expected to provide some benefit for maize production in water-limited regions[3]. For the C3 crops, wheat and rice, we calculated the CO_2-induced enhancement in yield for each of the six crop models, by subtracting the change in yield achieved with [CO_2] fixed at 2005 levels from the change when [CO_2] was allowed to evolve (Supplementary Table 3). These enhancements varied strongly between the models, although the median model response was similar to that expected based on FACE experiments. The effect on model projections was to give much smaller average yield declines for wheat and rice (Supplementary Figures 16, 17); non-water limited

yields were maintained, across much of the tropics, although even the most positive model (LPJ-GUESS) only projected small increases in non-water-limited yield in this region. CO_2 fertilisation may therefore alleviate some of the negative yield projections indicated by the analogue method, but we conclude that the knowledge of the effect of [CO_2] on crop yield is not yet far enough advanced to rely on this. Given its potential to limit the negative effects of climate change however, it must be a priority topic for future research, at least for C3 crops.

Supplementary Note 4

Baseline testing of the analogue method

In order to test the climate analogue method for a case of no change in mean climate we have repeated the calculations behind Fig. 1 using two random selections of 19 years from the period 1981-2010. Maps of the resulting yield changes show the "projected" yields to generally be within 10% of those from the training dataset (Supplementary Figure 20). Some areas of greater difference do exist, particularly for wheat, for which some marginal grid-cells (accounting for 0.6% of harvested area, but 3% of grid-cell area) are shifted towards higher attainable yields. This tendency to over-predict attainable yields in some marginal areas results from the drawing of analogues from regions which have access to irrigation sources disconnected from local irrigation (fossil groundwater, large rivers). The implications of this are discussed in the main text.

Supplementary References

1. Mueller, N. D. *et al.* Closing yield gaps through nutrient and water management. *Nature* **490,** 254–257 (2012).
2. Bondeau, A. *et al.* Modelling the role of agriculture for the 20th century global terrestrial carbon balance. *Glob. Chang. Biol.* **13,** 679–706 (2007).
3. Rosenzweig, C. *et al.* Assessing agricultural risks of climate change in the 21st century in a global gridded crop model intercomparison. *Proc. Natl. Acad. Sci. U. S. A.* **111,** 3268–73 (2014).
4. Tai, A. P. K., Martin, M. V. & Heald, C. L. Threat to future global food security from climate change and ozone air pollution. *Nat. Clim. Change* **4,** 817-821 (2014).
5. Elliott, J. *et al.* The Global Gridded Crop Model intercomparison: data and modeling protocols for Phase 1 (v1.0). *Geosci. Model Dev.* **8,** 261-277 (2015).
6. Olesen, J. E. *et al.* Uncertainties in projected impacts of climate change on European agriculture and terrestrial ecosystems based on scenarios from regional climate models. *Clim. Change* **81,** 123–143 (2007).
